# Infectious aetiologies of neonatal illness in south Asia classified using WHO definitions: a primary analysis of the ANISA study

**DOI:** 10.1016/S2214-109X(22)00244-3

**Published:** 2022-08-09

**Authors:** Melissa L Arvay, Nong Shang, Shamim A Qazi, Gary L Darmstadt, Mohammad Shahidul Islam, Daniel E Roth, Anran Liu, Nicholas E Connor, Belal Hossain, Qazi Sadeq-ur Rahman, Shams El Arifeen, Luke C Mullany, Anita K M Zaidi, Zulfiqar A Bhutta, Sajid B Soofi, Yasir Shafiq, Abdullah H Baqui, Dipak K Mitra, Pinaki Panigrahi, Kalpana Panigrahi, Anuradha Bose, Rita Isaac, Daniel Westreich, Steven R Meshnick, Samir K Saha, Stephanie J Schrag

**Affiliations:** a Division of Bacterial Diseases, Centers for Disease Control and Prevention, Atlanta, GA, USA; b Department of Epidemiology, UNC–Chapel Hill, Chapel Hill, NC, USA; c Department of Maternal Newborn Child and Adolescent Health, World Health Organization, Geneva, Switzerland; d Department of Pediatrics, Stanford University School of Medicine, Stanford, CA, USA; e Child Health Research Foundation, Dhaka, Bangladesh; f Department of Pediatrics, The Hospital for Sick Children, Toronto, ON, Canada; g Department of Clinical Research, London School of Hygiene & Tropical Medicine, London, UK; h International Centre for Diarrhoeal Disease Research, Bangladesh, Dhaka, Bangladesh; i Department of International Health, Johns Hopkins Bloomberg School of Public Health, Baltimore, MD, USA; j Department of Paediatrics and Child Health, Aga Khan University, Karachi, Pakistan; k Center of Excellence in Women and Child Health, Aga Khan University, Karachi, Pakistan; l Department of Pediatrics, Georgetown University Medical Center, Washington, DC, USA; m AIPH University, Bhubaneswar, India; n Christian Medical College, Vellore, India

## Abstract

**Background:**

Globally, neonatal mortality accounts for almost half of all deaths in children younger than 5 years. Aetiological agents of neonatal infection are difficult to identify because the clinical signs are non-specific. Using data from the Aetiology of Neonatal Infections in south Asia (ANISA) cohort, we aimed to describe the spectrum of infectious aetiologies of acute neonatal illness categorised post-hoc using the 2015 WHO case definitions of critical illness, clinical severe infection, and fast breathing only.

**Methods:**

Eligible infants were aged 0–59 days with possible serious bacterial infection and healthy infants enrolled in the ANISA study in Bangladesh, India, and Pakistan. We applied a partial latent class Bayesian model to estimate the prevalence of 27 pathogens detectable on PCR, pathogens detected by blood culture only, and illness not attributed to any infectious aetiology. Infants with at least one clinical specimen available were included in the analysis. We assessed the prevalence of these aetiologies according to WHO's case definitions of critically ill, clinical severe infection, and infants with late onset, isolated fast breathing. For the clinical severe definition, we compared the prevalence of signs by bacterial versus viral aetiology.

**Findings:**

There were 934 infants (992 episodes) in the critically ill category, 3769 (4000 episodes) in the clinical severe infection category, and 738 (771 episodes) in the late-onset isolated fast breathing category. We estimated the proportion of illness attributable to bacterial infection was 32·7% in infants in the critically ill group, 15·6% in the clinical severe infection group, and 8·8% among infants with late-onset isolated fast breathing group. An infectious aetiology was not identified in 58–82% of infants in these categories. Among 4000 episodes of clinical severe infection, those with bacterial versus viral attribution had higher proportions of hypothermia, movement only when stimulated, convulsions, and poor feeding.

**Interpretation:**

Our modelled results generally support the revised WHO case definitions, although a revision of the most severe case definition could be considered. Clinical criteria do not clearly differentiate between young infants with and without infectious aetiologies. Our results highlight the need for improved point-of-care diagnostics, and further study into neonatal deaths and episodes with no identified aetiology, to ensure antibiotic stewardship and targeted interventions.

**Funding:**

The Bill and Melinda Gates Foundation.

## Introduction

Neonatal mortality accounts for almost half of all deaths in children younger than 5 years worldwide.^1^, ^2^, ^3^, ^4^, ^5^, ^6^ More than a third of neonatal deaths globally occur in Bangladesh, India, and Pakistan; severe infections are among the leading causes.^7^ The aetiological agents of neonatal infections are difficult to identify in any setting because of non-specific signs; however, diagnosis is particularly challenging in low-resource settings as a result of restricted diagnostic capabilities. In these low-resource settings, young infants (aged 0–59 days) who are ill are often assessed by first-level health facility workers using WHO's Integrated Management of Childhood Illnesses (IMCI) clinical algorithm to identify those needing treatment or referral.^8^ Although this strategy has been widely adopted in low-resource settings, it is still common for the families of seriously ill young infants to decline to accept referral advice in some countries.^9^, ^10^, ^11^ Conversely, some infants who are not seriously ill might be hospitalised and receive parenteral antibiotic therapy unnecessarily.^12^

In response to challenges in the implementation of WHO's IMCI recommendations to treat infants with possible serious bacterial infection with 7 days of parenteral antibiotics in the hospital—as well as new evidence indicating that simplified antibiotic treatment is effective for non-critically ill infants when referral is refused^13^, ^14^, ^15^—WHO published revised guidelines in 2015 for management of possible serious bacterial infection when referral is not feasible.^8^ Simpler antibiotic regimens are included in the updated guidance and are defined as fewer injections (ranging from 2 to 7 days), followed by 5–7 days of oral antibiotics. The IMCI's case definition for possible serious bacterial infection was designed to be sensitive rather than specific, to minimise missed opportunities for life-saving treatment. The 2015 WHO guidelines further categorise young infants aged 0–59 days meeting the possible serious bacterial infection definition by severity, and provide treatment recommendations tailored to the severity level. Young infants aged at least 3 days with fast breathing as their only sign of illness—and thus considered to have the least severe disease—are recommended to receive oral antibiotic treatment in an outpatient setting, based on demonstrated effectiveness in this subgroup.^16^ Infants with clinical severe infection, which is the case definition most similar to the original 1996 of possible serious bacterial infection, are recommended to receive the simpler antibiotic regimens described above when referral is not feasible. Lastly, the most severely ill young infants, referred to as critically ill, are recommended to be referred to hospital for parenteral antibiotics and other supportive therapy after receiving a pre-referral dose of antibiotics.^8^ Much of the evidence used to inform this 2015 guideline was graded as low quality by WHO;^8^ a key gap identified in the data was scarce linkage between infectious aetiologies, clinical case definitions, and clinical management recommendations.

**Research in context**

**Evidence before this study**

We searched PubMed and OVID databases for relevant studies published in English between database inception and May 5, 2016, using 51 combinations of keywords, including “neonatal infections”, “south Asia”, “sepsis”, “young infant”, “aetiology”, “low income countries”, “clinical algorithm”, and “surveillance” as leading keywords. Our search yielded 317 references relevant to the research question. Clinical algorithms are useful to identify neonates with severe infection, particularly in settings where laboratories are not readily available. A 1999 community-based sepsis study conducted in rural India used the simultaneous presence of any two of seven clinical signs. These indicators predicted presumed sepsis death in neonates aged less than 1 month with a sensitivity of 100% and specificity of 92%. The criteria identified 10·6% of the neonates in the community with suspected cases of sepsis. The Young Infants Clinical Signs Study Group in 2008 evaluated infants (aged 0–6 days and 7–59 days) in outpatient health facilities and used the presence of any one of seven clinical signs to predict an expert paediatrician panel's clinical judgement of severe illness, requiring referral to a hospital for admission. The prediction model had a sensitivity of 85% and specificity of 75%. More recently, studies with standardised protocols have tested the use of simplified antibiotic regimens that can be provided in low-resource settings where referral to an inpatient facility or hospital might not be possible. Three of these trials (SAT-Bangladesh, SAT-Pakistan, and AFRINEST-severe infections) studied whether young infants with clinical signs indicative of possible severe infection can be treated with a combination of oral amoxicillin plus gentamicin injections, and whether injections can be stopped after the first 2 days to make treatment regimens simpler. These equivalency trials examined whether adherence to regimens differs between simpler antibiotic regimens and the standard regimen of procaine penicillin and gentamicin injections daily for 7 days, using a predefined margin of ±5%. These data provided some evidence for the 2015 revision of WHO's Integrated Management of Childhood Illnesses guidelines for managing possible serious bacterial infection in young infants when referral is not possible. Much of the evidence used to inform these guidelines was graded as low quality, and comprehensive diagnostic evaluation of infants in the different case strata was missing, preventing a link between infectious aetiologies and clinical management recommendations being established. Most studies have been conducted in high-income countries and have focused on early-onset neonatal sepsis and, more specifically, group B *Streptococcus*.

**Added value of this study**

Our study suggests that infants are probably being treated unnecessarily with antibiotics in low-income or low-resource settings given that they meet the case definition for such treatment, but might have a viral aetiology or no infectious aetiology at all. As such, there could be a substantial proportion of illness for which other interventions would be more effective, or there might be several unidentified pathogens causing illness. Conversely, although the proportion of infants with a bacterial aetiology was highest in the most severe category and lowest in the least severe category, there was still a non-zero proportion of those with bacterial aetiology in every category. The findings from our analysis also suggest it would be difficult to develop a clinical algorithm to distinguish bacterial from viral infections.

**Implications of all the available evidence**

If the trials conducted in Africa and south Asia that evaluated the equivalency of simpler antibiotic regimens to the conventional regimens had restricted their study population to those with a bacterial aetiology, simplified regimens might not have performed as well. However, in the absence of a method available to identify the aetiology of illness more readily in low-resource settings, this analysis supports the current set of case definitions and the 2015 WHO guideline as appropriate tools for referral and treatment among those refusing referral, with a possibility for further refinement of the critical illness case definition. These results highlight the continued need for improved diagnostics, as they are key to further understanding the causes of illness in neonates. Continued investigation into infants who died, and those infants with no aetiological attribution, is needed to develop targeted interventions. From a methodological perspective, better diagnostics for Bayesian partial latent class model methods could be developed to ensure precise and unbiased estimates are generated using these methods in a range of public health scenarios.

The Aetiology of Neonatal Infections in south Asia (ANISA) study was a multicentre, population-based longitudinal study done in 2010–14 that aimed to determine the population-based incidence, aetiology, and antibiotic resistance profiles of community-acquired infections among young infants aged 0–59 days in Bangladesh, India, and Pakistan.^17^ The primary aetiological analysis of this cohort of more than 5000 infants born in south Asia who met the possible serious bacterial infection case definition used diagnostic testing results available for bacteria and viruses in blood and respiratory samples, and data from matched healthy infants in the same communities. Aetiological causes were attributed to 28% of all episodes.

In this analysis of the ANISA cohort, we aimed to describe the spectrum of infectious aetiologies of acute neonatal illness categorised post-hoc using the 2015 WHO case definitions of critical illness, clinical severe infection, and fast breathing only (in the late-onset period of 3–59 days post-birth), and evaluate each definition's ability to identify young infants with bacterial infection.^17^ We also aimed to describe the proportion of clinical severe infection in infants attributable to bacterial or viral infection, by individual IMCI clinical signs, to determine whether there are clinical signs predictive of bacterial infection.

## Methods

### Study design and population

The ANISA study enrolled married girls and women of childbearing age (aged 13–49), in defined catchment areas, who were followed up for pregnancy and birth outcomes. Each infant born to an enrolled woman was assessed for illness at ten scheduled household visits during the first 59 days of life, using the IMCI possible serious bacterial infection case definition, and managed according to WHO guidelines for the management of possible serious bacterial infection that were current in 2011.^12^, ^17^ All study personnel underwent training to ensure assessment of ill children was standardised across the five study sites (Sylhet, Bangladesh; Matiari and Karachi, Pakistan; and Vellore and Odisha, India).

Respiratory (nasopharyngeal and oropharyngeal swabs) and blood specimens were collected from infants with possible serious bacterial infection to test for the presence of pathogens.^18^ A subset of healthy infants (n=1895) was also enrolled in the ANISA study^17^ to provide a comparison group of asymptomatic carriage of bacteria and viruses. These healthy infants were matched to ill infants on the basis of age, were selected at the same time as the ill infant presented symptoms, and were visited at home (or at clinics) to collect respiratory and blood specimens ten times during the first 59 days of life. These specimens from healthy infants were used to obtain the background carriage rate, and were used as priors to inform the false positive rate in the partial latent class model. No epidemiological or clinical data collected from healthy infants informed this analysis. Blood specimens were tested using automated blood culture methods (for ill infants only) to detect bacteria, and blood and respiratory specimens underwent PCR TaqMan Array card panel testing for 27 different bacteria and viruses (appendix p 1). The study surveillance and laboratory methods have been described previously.^17^, ^18^, ^19^, ^20^

For the ANISA study,^17^ informed verbal consent was obtained from study participants when they were registered in the study, and written informed consent was obtained from parents or caregivers when samples were collected from babies. The study was approved by the ethics committees or internal review boards of all participating organisations. The US Centers for Disease Control and Prevention relied on all local institutional review boards for ethical review.

### Case definitions

We defined an infant with clinical severe infection as one who presented with at least one of the following signs: severe chest in-drawing, atypical axillary temperature (≥38°C or <35·5°C), movement only when stimulated, and failure to feed well (confirmed on observation by study personnel).^11^ We defined a critically ill infant as one who presented with at least one of the following signs: absence of consciousness (no movement at all), inability to feed, inability to cry, physician-observed convulsions, apnoea, cyanosis, bulging fontanelle, major congenital malformations inhibiting oral antibiotic intake, and persistent vomiting (defined as vomiting after three attempts to feed within 30 min). We defined an infant with late-onset isolated fast breathing as one aged 3–59 days with an elevated respiratory rate (ie, ≥60 breaths per min) as their only sign. Each of these case definitions are mutually exclusive. All clinical signs used in these definitions were based on physicians’ assessments.

### Statistical analysis

A partial latent class model was used to estimate pathogen population proportions for infants with clinical severe infection and compare these proportions to those for infants who were critically ill and those with isolated late-onset fast breathing. This method was chosen for its ability to incorporate multiple tests per pathogen for a given case into the estimation of pathogen probabilities at an individual and population level, and allowed us to quantify the uncertainty around diagnostic tests.^17^, ^21^, ^22^ Infants with at least one clinical specimen available were included in the analysis (ANISA and our aetiological analyses). For the analysis including infants with isolated fast breathing, we used the same true-positive rates as estimated by the ANISA aetiology model; we set these values as constants, therefore they were not estimated by our model. Statistical differences between proportions are defined as statistically significant p values (≤0·05) derived from single or multiple χ^2^ tests, or by overlapping Bayesian 95% credible intervals (CrIs). We looked for patterns of missingness in the ANISA data,^17^ and did not find any discernible patterns of non-random missingness. The comparison group for the critically ill infants consisted of all infants with clinical severe infection, while the comparison group for the infants with late-onset isolated fast breathing was restricted to infants (aged 3–59 days) with clinical severe infection. Our analysis of infants with isolated fast breathing was restricted to the Bangladesh site because this was the only site where respiratory and blood specimens were routinely collected from such infants. We hypothesised that the proportion of illness attributed to bacteria would be higher in critically ill infants than in those with clinical severe infection, and that infants with isolated fast breathing in the late-onset period would have lower proportions of bacterial aetiologies than those in the same age group with clinical severe infection.

#### Aetiological attribution

ANISA statistical methodology is an extension of the basic partially latent class model developed for the Pneumonia Etiology Research for Child Health study to estimate the proportion of pneumonia infections attributed to one of 33 specific pathogen classes.^23^ We applied the same partial latent class Bayesian model as used in the ANISA study, which used a Gibbs sampler to estimate individual-level and population-level probabilities for each of the 27 pathogens detectable on the molecular PCR TaqMan Array card platform, and an other (or none) category, which included cases not attributed to any aetiology.^17^, ^22^ The Gibbs sampler is a Markov Chain Monte Carlo algorithm for obtaining a sequence of observations that approximate a specified multivariate probability distribution. Each sequence was used to estimate the latent variables of aetiology proportion and test characteristics—ie, the true-positive rate and false-positive rate for every test and pathogen. The detections in specimens obtained from healthy infants were used to set priors for the false-positive rates in the model. Another class was indirectly calculated (ie, not estimated by the model), for all other bacteria that were detectable by blood culture but did not have a corresponding molecular test. The mean, 2·5th, and 97·5th percentiles for the Bayesian CrIs are reported.

#### Distribution of clinical signs among cases with infectious aetiology

To examine the distribution of clinical signs among infants who met the clinical severe infection case definition and had an infectious aetiology, we used the clinical severe infection model output, which generates an estimated probability for each of the 27 pathogens examined. We calculated the posterior probability for every clinical sign in the IMCI case definition using the standard Bayesian formula.^24^ We used the posterior probabilities from every iteration to obtain a distribution for each clinical sign, and reported the median as the point estimate and 2·5th and 97·5th percentiles for the Bayesian CrIs. We stratified these probabilities by early onset (infants aged <3 days) and late onset (infants aged ≥3–59 days) of disease. Within these categories, we further stratified by aetiology: bacterial versus viral.

### Role of the funding source

The funder of the study had no role in study design, data collection, data analysis, data interpretation, or writing of the report.

## Results

There were 934 infants (992 episodes) in the critically ill category, 3769 (4000 episodes) in the clinical severe infection category, and 738 (771 episodes) in the late-onset isolated fast breathing category. Infants meeting the critically ill case definition and the clinical severe infection case definition were more likely to have been born preterm or at low birthweight than were infants with late-onset isolated fast breathing (table 1). Critically ill infants were significantly more likely to be hospitalised (48·8%) or die within 7 days of illness onset (18·2%) than infants who met the clinical severe infection case definition (31·8% hospitalised, 3·7% died within 7 days) and those with isolated fast breathing (14·5% hospitalised, 1·6% died within 7 days). ANISA only captured clinical and diagnostic data on 9% of infants who died within 3 days of illness onset.^17^ Among the 9% of deaths for which a physician assessment was performed, respiratory and blood specimens were collected from 22·9% of those in the critically ill group, 9·5% of infants in the clinical severe infection group, and 2·1% of those in the isolated fast breathing group. There was a low proportion of cases with missing data (<5%), and we assumed that these data were missing at random.

**Table 1:** Characteristics of infants presenting with possible serious bacterial infection case definitions,^11^ 2011–14

		**Critically ill infants (n=934)**	**Infants with clinical severe infection (n=3769)**	**Infants with late-onset isolated fast breathing (n=738)**	**p value**
Sex					
	Male	545 (58·3%)	2114 (56·1%)	441 (59·8%)	0·56*
	Female	389 (41·7%)	1655 (43·9%)	297 (40·2%)	
Preterm (<37 weeks' gestation)		268 (28·7%)	1050 (27·9%)	132 (17·9%)	<0·0001*
Low birthweight (<2500 g)		357 (38·2%)	1373 (36·4%)	185 (25·1%)	<0·0001*
Site					
	Sylhet, Bangladesh	302 (32·3%)	873 (23·2%)	738 (100%)	<0·0001
	Karachi, Pakistan	102 (10·9%)	1065 (28·3%)	..	<0·0001
	Matiari, Pakistan	171 (18·3%)	997 (26·5%)	..	<0·0001
	Vellore, India	29 (3·1%)	409 (10·9%)	..	<0·0001
	Odisha, India	330 (35·3%)	425 (11·3%)	..	<0·0001
Episodes of possible serious bacterial infection		992	4000	771	..
	Early onset†	508 (51·2%)	2448 (61·2%)	0	<0·0001
Integrated management of childhood infection signs					
	Respiratory rate ≥60 breaths per minute	375 (37·8%)	1580 (39·5%)	771 (100%)	0·33
	Severe chest in-drawing	153 (15·4%)	1086 (27·2%)	0	<0·0001
	Axillary temperature ≥38·0°C (>100·4°F)‡	229 (23·1%)	1608 (40·2%)	0	<0·0001
	Axillary temperature <35·5°C (<95·9°F)‡	157 (15·8%)	377 (9·4%)	0	<0·0001
	Movement only when stimulated	307 (31·0%)	332 (8·3%)	0	<0·0001
	No movement at all (unconscious)	44 (4·4%)	0	0	..
	Convulsions§	223 (22·5%)	0	0	..
	Poor feeding§	680 (68·6%)	1455 (36·4%)	0	<0·0001
Other signs					
	Bulging fontanelle	40 (4·0%)	0	0	..
	Persistent vomiting	64 (6·5%)	0	0	..
	Unable to cry	306 (30·9%)	0	0	..
	Presence of apnoea	92 (9·3%)	0	0	..
	Presence of cyanosis	314 (31·7%)	0	0	..
Child hospitalised		484 (48·8%)	1562 (31·8%)	112 (14·5%)	<0·0001*
Child died¶		227 (22·9%)	251 (9·5%)	16 (2·1%)	<0·0001*
Child died within 7 days of episode¶		181 (18·2%)	146 (3·7%)	12 (1·6%)	<0·0001*

Data are n (%), unless otherwise stated, obtained from the ANISA study.^17^ Critically ill infants presented with at least one of the following signs: absence of consciousness (no movement at all), inability to feed, inability to cry, physician-observed convulsions, apnoea, cyanosis, bulging fontanelle, major congenital malformations inhibiting oral antibiotic intake, and persistent vomiting (defined as vomiting following three attempts to feed within 30 min). Infants with clinical severe infection presented with at least one of the following signs: severe chest in-drawing, atypical axillary temperature (≥38°C or <35·5°C), movement only when stimulated, and failure to feed well (confirmed on observation by study personnel). Late-onset isolated fast breathing is defined as an infant aged 3–59 days with elevated respiratory rate (ie, ≥60 per min) as their only sign of illness. These infants were enrolled in the Bangladesh site only.

* Multiple χ^2^ comparisons.

† Early onset is defined as onset during the first 3 days post-birth.

‡ Temperature was measured in Fahrenheit.

§ Confirmed by observation.

¶ Deaths with clinical information available are presented.

**Figure 1:**
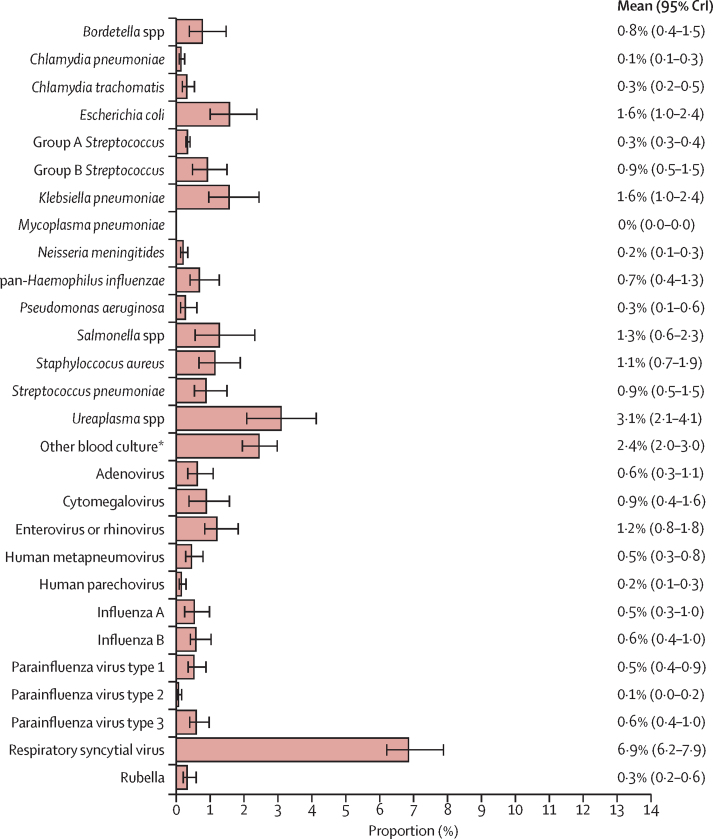
Estimates from a partial latent class model of the prevalence of pathogens in infants meeting the clinical severe infection case definition (n=4000) Data are from the ANISA study, 2011–14.^17^ Error bars are 95% CrI. Proportion in the other or none category: 71·7% (68·9–74·5), which includes any possible serious bacterial infection episode that was not attributed by the partial latent class model to one of the pathogen classes in ANISA. CrI=credible interval. *Includes all bacteria that grew on blood culture but did not have an associated assay on the ANISA molecular diagnostic panel. The full list of pathogens isolated can be found in the appendix (pp 1–2). This category was not estimated by the model directly.

Among infants meeting the case definition for clinical severe infection, the most commonly identified pathogens were respiratory syncytial virus (6·9%; 95% CrI 6·2–7·9), *Ureaplasma* spp (3·1%; 2·1–4·1), and other bacterial pathogens not tested for by TaqMan Array card (2·4%; 2·0–3·0). A large proportion of the population with clinical severe infection (71·7%; 68·9–74·5) did not have an aetiological attribution (figure 1).

**Figure 2:**
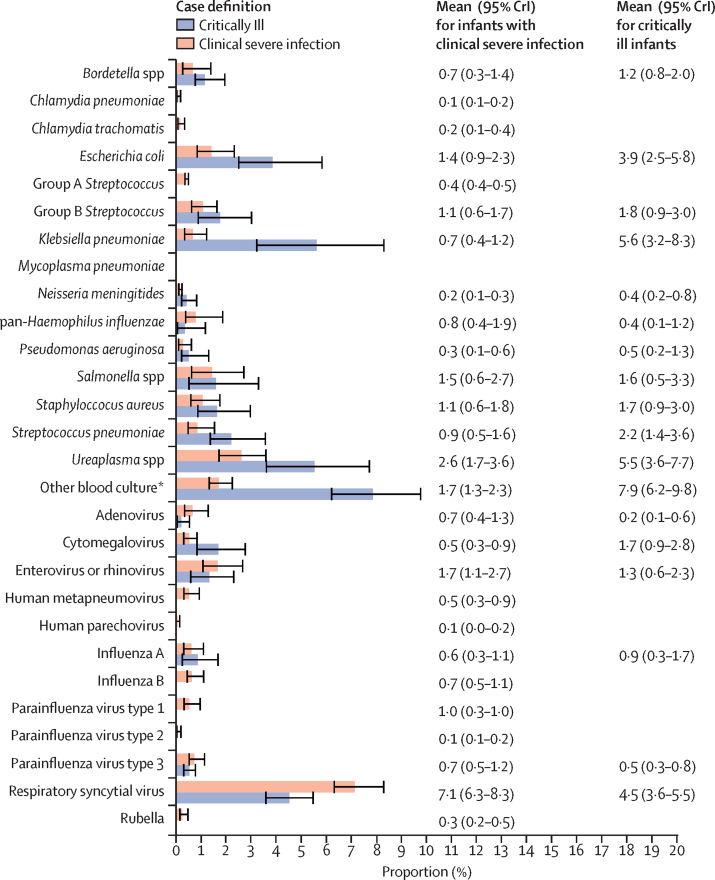
Estimates from a partial latent class model of the prevalence of pathogens in infants meeting the critically ill (n=992) or clinical severe infection (n=4000) case definitions Data are from the ANISA study, 2011–14.^17^ Error bars are 95% CrI. Proportions in the other or none category were 58·2% (51·8–63·7) for critically ill infants and 72·9% (69·6–75·7) for infants with clinical severe infection, which includes any possible serious bacterial infection episode that was not attributed by the partial latent class model to one of the pathogen classes in ANISA. CrI=credible interval. *Includes all bacteria that grew on blood culture but did not have an associated assay on the ANISA molecular diagnostic panel. The full list of pathogens isolated can be found in the appendix (pp 1–2). This category was not estimated by the model directly.

Among critically ill infants, the most commonly identified pathogens were other bacterial pathogens not tested for by TaqMan Array card (7·9%; 95% CrI 6·2–9·8), *Klebsiella pneumoniae* (5·6%; 3·2–8·3), *Escherichia coli* (3·9%; 2·5–5·8), and *Ureaplasma s*pp (5·5%; 3·6–7·7; figure 2).

A comparison of episodes of illness attributable to pathogens in the critically ill group versus the clinical severe infection group revealed more illness attributable to bacteria in the critically ill (32·7%) group than in the clinical severe infection group (15·6%). A higher proportion of critically ill case episodes were attributed to any infectious aetiology (41·8%; 36·3–48·2) than were clinical severe infection case episodes (27·1%; 24·3–30·4).

**Figure 3:**
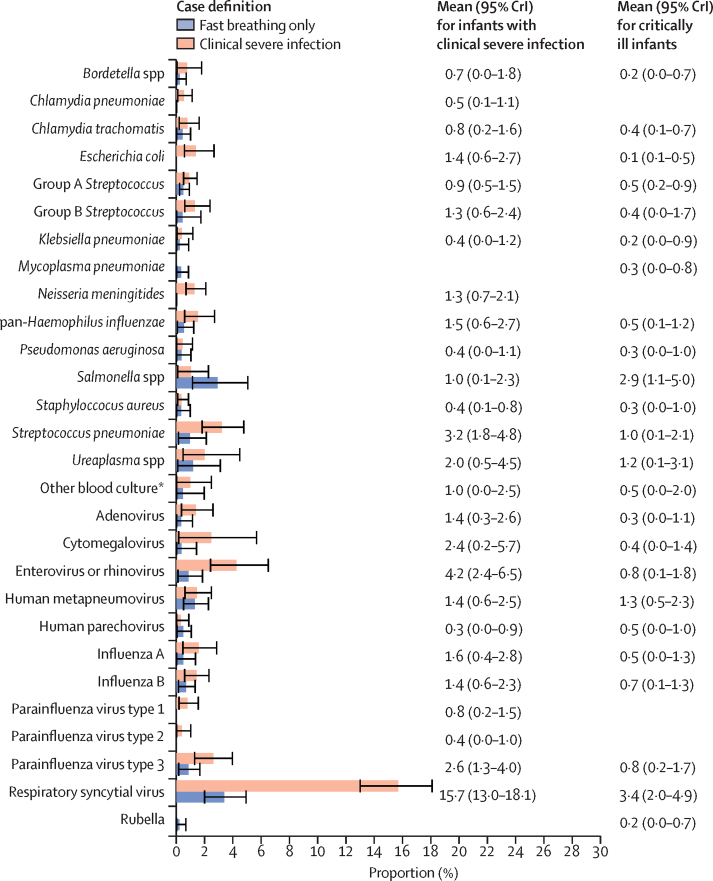
Estimates from a partial latent class model of the prevalence of pathogens in infants presenting with isolated fast breathing (n=771) and those meeting the clinical severe infection case definition (n=1552), late onset of illness (3–59 days post-birth) Data are from the ANISA study, 2011–14.^17^ Error bars are 95% CrI. Our analysis of infants with isolated fast breathing was restricted to the Bangladesh site (Sylhet) because this was the only site where respiratory and blood specimens were routinely collected from such infants. Proportions in the other or none category were 82·5% (78·7–86·0) for infants with late-onset fast breathing only and 51·3% (45·7–56·9) for infants with clinical severe infection, which includes any possible serious bacterial infection episode that was not attributed by the partial latent class model to one of the pathogen classes in ANISA. CrI=credible interval. *Includes all bacteria that grew on blood culture but did not have an associated assay on the ANISA molecular diagnostic panel. The full list of pathogens isolated can be found in the appendix (p 3). This category was not estimated by the model directly.

Overall, 9% of late-onset isolated fast breathing cases had an attributable bacterial aetiology (figure 3). respiratory syncytial virus was the leading pathogen among infants with late-onset isolated fast breathing (3·4%; 95% CrI: 2·0–4·9) and those with clinical severe infection (15·7%; 13·0–18·1). There were significantly higher proportions of *E coli* (1·4% [0·6–2·7] *vs* 0·1% [0·1–0·5]) and enterovirus or rhinovirus (4·2% [2·4–6·5] *vs* 0·8% [0·1–1·8]) in the clinical severe infection group than in the late-onset isolated fast breathing group. A significantly higher proportion (82·5%; 78·7–86·0) of the isolated fast breathing cases could not be attributed to any aetiology, as compared with 51·3% (45·7–59·9) of late-onset clinical severe infection cases.

A comparison of clinical presentation among infants with clinical severe infection and bacterial or viral aetiology showed several significant differences in proportions of clinical signs between infants with bacterial versus viral aetiology (table 2). Infants with bacterial aetiology had significantly higher proportions of hypothermia (12·2% [95% CrI 10·9–13·4] *vs* 3·7% [2·9–4·4]), movement only when stimulated (18·6% [17·0–20·1] *vs* 6·5% [5·5–7·6]), convulsions (4·5% [4·0–5·1] *vs* 1·5% [1·2–1·9]), and poor feeding (47·9% [45·1–50·5] *vs* 26·1% [24·3–28·0]), than did infants with a viral aetiology. When further stratifying this comparison by early and late onset of illness there were significant differences in clinical presentation between those infants with early and late onset of disease (table 2). Among infants with early onset of disease, there was a significantly higher proportion of infants with movement only when stimulated or no movement (24·2% [21·8–26·6] *vs* 12·0% [6·2–18·2]) and poor feeding (67·3% [63·8–70·2] *vs* 54·7% [45·6–62·3]) in the bacterial aetiology group than in the viral aetiology group. Among infants with late-onset disease, the bacterial aetiology group had significantly higher proportions of every clinical sign except for fast breathing and severe chest in-drawing, which were higher in the viral aetiology group. When comparing the bacterial aetiology groups across early and late onset of disease, there were significantly higher proportions of every clinical sign in the early onset group, except for severe chest in-drawing and fever, which were significantly higher in the late-onset group.

**Table 2:** Distribution of signs among infants meeting the clinical severe infection case definition (n=4000), by viral or bacterial aetiology and age of onset, 2011–14

	**Bacterial aetiology**	**Viral aetiology**	**Early onset**		**Late onset**	
			Bacterial aetiology	Viral aetiology	Bacterial aetiology	Viral aetiology
Respiratory rate ≥60 breaths per minute	37·3% (35·7–38·8)	42·4% (41·2–43·6)	41·8% (39·7–43·8)	38·4% (32·2–44·4)	34·5% (32·6–36·5)	42·7% (41·4–44·0)
Severe chest in-drawing	22·0% (19·8–24·3)	51·1% (48·6–53·6)	9·6% (8·5–10·7)	5·7% (3·1–9·7)	29·6% (26·7–32·6)	54·2% (51·9–56·3)
Axillary temperature >38·0°C (>100·4°F)*	32·9% (31·2–34·9)	32·8% (31·1–34·4)	26·1% (23·3–29·4)	29·5% (21·0–40·5)	37·1% (35·2–38·9)	33·0% (31·5–34·5)
Axillary temperature <35·5°C (<95·9°F)*	12·2% (10·9–13·4)	3·7% (2·9–4·4)	19·8% (17·8–21·8)	24·0% (17·7–30·2)	7·5% (6·4–8·5)	2·3% (1·9–2·7)
Movement only when stimulated or no movement	18·6% (17·0–20·1)	6·5% (5·5–7·6)	24·2% (21·8–26·6)	12·0% (6·2–18·2)	15·2% (13·7–16·7)	6·1% (5·2–7·1)
Convulsions†	4·5% (4·0–5·1)	1·5% (1·2–1·9)	8·4% (7·3–9·5)	5·6% (2·3–9·2)	2·1% (1·8–2·5)	1·2% (1·0–1·4)
Poor feeding†	47·9% (45·1–50·5)	26·1% (24·3–28·0)	67·3% (63·8–70·2)	54·7% (45·6–62·3)	36·0% (33·2–38·9)	24·2% (22·4–25·9)

Data are % (95% credible interval), obtained from the ANISA study.^17^ Early onset is defined as onset during the first 3 days post-birth. Late onset is defined as onset between 3–59 post-birth.

* Temperature was measured in Fahrenheit.

† Confirmed by observation.

## Discussion

At the time the original IMCI guidelines were written, scarce aetiological evidence was available to inform the guidelines for management of illness in young infants; in particular, the studies available were restricted by small sample sizes and blood culture as the only diagnostic test.^25^, ^26^, ^27^ The ANISA study is the first of its kind to characterise the aetiological spectrum in young infants presenting with possible serious bacterial infection in a large, prospective, multisite cohort study in settings where referral is frequently declined. Our analysis further described the spectrum of infectious aetiologies of acute neonatal illness according to WHO's case definitions for critical illness, clinical severe infection, and fast breathing only, and evaluated the ability of each definition to identify young infants with bacterial infection.

Infants meeting the critically ill case definition had a higher proportion of bacterial infections (32·7%) than infants in the clinical severe infection group (15·6%), which warrants continued reinforcement that referral is imperative for critically ill infants. The data for young infants with late-onset isolated fast breathing generally support the revised guideline, which recommends treatment of infants in this category with outpatient oral antibiotics. Although 18% of possible serious bacterial infections in this group were attributed to any infectious aetiology, half of these were attributed to viral infection.

Notably, although the aetiological data support recent WHO distinctions in clinical presentation, there is still a large proportion of unexplained illness in all three groups of infants in our analysis, despite testing for 28 pathogen classes. It is possible that specimens included in the ANISA study analysis were not adequate to detect all infections (eg, no cerebrospinal fluid or urine), or a pathogen contributing to the unexplained aetiology group was not included in the test panel. However, it is also plausible that many episodes of possible serious bacterial infection have non-infectious causes, such as intrapartum events or congenital conditions.

Although the proportion of infants with a bacterial aetiology was highest in the most severe category, and lowest in the least severe category, there was still a non-zero proportion of infants with bacterial aetiology in every category. If the trials conducted in Africa and south Asia that evaluated the equivalency of simpler antibiotic regimens to the conventional regimens^13^, ^14^, ^15^ had restricted their study population to those with a bacterial aetiology, simplified regimens might not have performed as well.

Given that most young infants in the ANISA cohort had an unexplained illness aetiology, we further examined the clinical presentation of infants meeting the most commonly observed clinical severe infection definition to identify clinical signs associated with bacterial infections, as opposed to viral infections. Infants in the clinical severe infection group with a bacterial aetiology had significantly higher proportions of the clinical signs typically associated with more severe infection (such as hypothermia, convulsions, and movement only with stimulation); therefore, restricting use of these clinical signs to the critically ill case definition could be considered.

Research suggests that biomarkers (such as C-reactive protein and certain toll-like receptors^28^), and new metagenomic sequencing to rapidly identify pathogens from normally sterile fluids, could help to diagnose infection. New neonatal sepsis predictive algorithm tools have also been developed,^29^, ^30^, ^31^ but it is unlikely that physicians would have this information or technology available in low-resource public health settings.

Although currently recommended treatment regimens target most of the bacteria identified in infants in all three illness categories defined by WHO, *Ureaplasma* represents a comparatively high proportion of infection in all groups. *Ureaplasma* has not been identified previously as a relevant pathogen for term infants in this age group, and the currently recommended regimens (typically gentamicin or amoxicillin) would not be effective against this agent. Regimens used to treat *Ureaplasma* infection typically include macrolides or tetracyclines.^32^ In the most severely ill infants in the ANISA study, 5·5% of episodes would not have been treated with antibiotics that are effective against the aetiological agent of illness.

This analysis has several limitations. Our study population is restricted to infants who met the WHO IMCI case definition for possible serious bacterial infection. Ideally, we would run a predictive model to measure which clinical signs best predict the outcome of each infectious aetiology; however, this approach is not possible because the outcome itself is defined by the clinical signs. As in the main ANISA aetiology study, we set no bounds on the true-positive rate for the blood culture sensitivity. Combined with the small sample size, this setting of no bounds on the true-positive rate could result in the estimated proportion for certain pathogens being inflated. There are other criteria included in the critically ill case definition (major congenital malformations inhibiting oral antibiotic intake) that were not recorded by the ANISA study and, therefore, were not evaluated. Although considerable effort was made to reach infants on day zero of their lives, there were many severely ill infants who went on to die who were missed on the first hours of life. Hence, the ANISA study^17^ was only able to collect clinical specimens on 9% of all deaths. Aetiological attribution is challenging because infants often have multiple results for many pathogens. Although the partially latent class model method allows for the use of multiple test results to be used in the estimation of aetiology proportions, there are certain circumstances and conditions when using the partially latent class model methods—such as low number of positive detections, or having only a single test available for a given pathogen—where estimation of the true aetiology proportion has low or restricted reliability. The limit of detection was low for many of the assays on the blood panel, and there were few positive blood cultures within the study population. Several strategies described in the methods of the ANISA study,^17^, ^22^ such as the incorporation of multiple specimen diagnostic results and test characteristics into the model, help to mitigate this issue.^22^ An additional limitation is that our analysis of infants with isolated fast breathing was restricted to the Bangladesh site, given that this site was the only study location where respiratory and blood specimens were routinely collected from such infants.

The ANISA study was conducted in countries with relatively high burdens of neonatal disease and mortality, and high rates of caregivers declining referral for treatment or hospitalisation, thereby providing the appropriate population to describe the aetiological spectrum of disease in young infants. This analysis presents the first data that describe the aetiology of infections in young infants meeting all three WHO case definitions. Although infants are probably being overtreated with antibiotics in these settings, offering referral and treatment is warranted as there are infectious aetiologies among even the least severely ill infants. Conversely, a substantial proportion of infants with illness meeting IMCI case definitions might have viral or non-infectious aetiologies—for which antibiotic treatment is unnecessary and other treatments are more effective—because standard clinical signs for possible serious bacterial infection overlap with other non-infectious conditions (such as congenital defects or intrapartum events). Further investigation into infants who died or had no identified infectious aetiology is needed, as are improved point-of-care diagnostics. These steps will help to ensure appropriate antibiotic stewardship and targeted interventions to prevent morbidity and mortality in this vulnerable group of infants.

## Data sharing

The data collected as part of the ANISA study^17^ were shared with all participating collaborators, but will not be made available to others.

## Declaration of interests

We declare no competing interests.
