# Correction to *Lancet Glob Health* 2022; 10: e1289–97

**DOI:** 10.1016/S2214-109X(22)00385-0

**Published:** 2022-08-30

*Arvay ML, Shang N, Qazi SA, et al. Infectious aetiologies of neonatal illness in south Asia classified using WHO definitions: a primary analysis of the ANISA study.* Lancet Glob Health *2022;* 10: *e1289–97*—In this Article, the Role of the funding source statement should have included the following: “AKMZ was the Principal Investigator for the Pakistan site at Aga Khan University during the study enrolment period (2010–12). She is currently President of Gender Equality and director of Enteric and Diarrheal Diseases and Vaccine Development at the Bill & Melinda Gates Foundation (BMGF), and an adjunct faculty member at the Aga Khan University. Her roles at BMGF have had no connection to this analysis. AKMZ led the original study design and data collection in Pakistan, and provided input to the primary author that the data interpretation and analysis in this manuscript were accurate. She approved the final submission.” This statement replaces a more brief statement previously in the Acknowledgments section. This correction has been made as of Aug 30, 2022.

