# Supplementary material for: Infectious aetiologies of neonatal illness in south Asia classified using WHO definitions: a primary analysis of the ANISA study
Source: Lancet Glob Health. 2022 Aug 9;10(9):e1289–97. doi: 10.1016/S2214-109X(22)00244-3 (PMC9380253; doi:10.1016/S2214-109X(22)00244-3)
Supplement: Supplementary appendix [file mmc1.pdf]

THE LANCET
Global Health

Supplementary appendix

This appendix formed part of the original submission and has been peer reviewed. We post it as supplied by the authors.

Supplement to: Arvay ML, Shang N, Qazi SA, et al. Infectious aetiologies of neonatal illness in south Asia classified using WHO definitions: a primary analysis of the ANISA study. *Lancet Glob Health* 2022; **10:** e1289–97.

Appendix 1

Table of Contents

Supplemental Material 1. Overall molecular polymerase chain reaction detections by Tacman Array Cards (TAC) of sepsis pathogens among infants with possible serious infection by case definition, Aetiology of Newborn Infections in South Asia (ANISA) Study, 2011-14……………………………….page 1

Supplemental material 2. Characteristics of pathogens isolated from blood culture among infants with possible serious infection, by critically ill and clinical severe infection status, Aetiology of Newborn Infections in South Asia (ANISA) Study, 2011-14………………………………………………………………..page 2

Supplemental material 3. Characteristics of pathogens isolated from blood culture among infants with possible serious infection in Sylhet, by age of onset and fast breathing only status, Aetiology of Newborn Infections in South Asia (ANISA) Study, 2011-14………………………………………………………………..page 3

Supplemental Material 1. Overall molecular polymerase chain reaction detections by Tacman Array Cards (TAC) of sepsis pathogens among infants with possible serious infection by case definition, Aetiology of Newborn Infections in South Asia (ANISA) Study, 2011-14

	Critically Ill[2]						Clinical severe infection[3]						Sylhet Fast Breathing Only[4]					
	NP/OP[1] TAC			Blood TAC			NP/OP TAC			Blood TAC			NP/OP TAC			Blood TAC		
	**Positive cases (%)**	**Positive healthy infants (%)**	**Odds Ratio**	**Positive cases (%)**	**Positive healthy infants (%)**	**Odds Ratio**	**Positive cases (%)**	**Positive healthy infants (%)**	**Odds Ratio**	**Positive cases (%)**	**Positive healthy infants (%)**	**Odds Ratio**	**Positive cases (%)**	**Positive healthy infants (%)**	**Odds Ratio**	**Positive cases (%)**	**Positive healthy infants (%)**	**Odds Ratio**
**Number tested**	**N = 977**	**N = 1893**		**N = 747**	**N = 1717**		**N = 3971**	**N = 1893**		**N = 3255**	**N = 1717**		**N = 1161**	**N = 412**		**N = 847**	**N = 344**	
**Pathogen**																		
Adenovirus	14 (1.4)	34(1.8)	0.8				58 (1.5)	34(1.8)	0.8				16 (1.4)	12(2.9)	0.5			
Bordetella sp.	25 (2.6)	73(3.9)	0.7				203 (5.1)	73(3.9)	1.3				12 (1.0)	6(1.5)	0.7			
Chlamydia pneumoniae	1 (0.1)	3(0.2)	0.5				9 (0.2)	3(0.2)	1.0				1 (0.9)	2(0.5)	1.8			
Chlamydia trachomatis	2 (0.2)	5(0.3)	0.7				10 (0.3)	5(0.3)	1.0				3 (0.3)	1(0.2)	1.5			
Cytomegalovirus	87 (9.4)	153(8.5)	1.1				245(6.8)	153(8.5)	0.8				82 (8.1)	44(12.3)	0.7			
Escherichia coli	336(34.4)	461(24.4)	1.4	19 (2.5)	32(1.9)	0.5	958 (24.1)	461(24.4)	1.0	52 (1.6)	32(1.9)	0.8	430(37.0)	123(29.9)	1.2	15 (1.8)	11(3.2)	0.6
Influenza A	12 (1.2)	13(0.7)	1.7				39 (1.0)	13(0.7)	1.4				10 (0.9)	3(0.7)	1.3			
Influenza B	2 (0.2)	3(0.2)	1.0				29 (0.7)	3(0.2)	3.5				6 (0.5)	0(0)	/			
Group A *Streptococcus*				2 (0.3)	0(0)	/				12 (0.4)	0(0)	/				3 (0.4)	0(0)	/
Group B *Streptococcus*	92 (9.4)	123(6.5)	1.4	7 (0.9)	6(0.4)	2.3	309 (7.8)	123(6.5)	1.2	11 (0.3)	6(0.4)	0.8	239 (20.6)	74(18.0)	1.1	4 (0.5)	6(1.7)	0.3
pan-*Haemophilus influenzae*				10 (1.3)	6(0.4)	3.3				25 (0.8)	6(0.4)	2.0				7 (0.8)	3(0.9)	0.9
Human metapneumovirus	1 (0.1)	6(0.3)	0.3				20 (0.5)	6(0.3)	1.7				11 (1.0)	1(0.2)	5.0			
Human parechovirus	2 (0.2)	12(0.6)	0.3				13 (0.3)	12(0.6)	0.5				7 (0.6)	5(1.2)	0.5			
Klebsiella pneumoniae	274(28.1)	422(22.3)	1.3	24 (3.2)	33(1.9)	1.7	817 (20.1)	422(22.3)	0.9	51 (1.6)	33(1.9)	0.8	308 (26.5)	101(24.5)	1.1	45 (5.3)	22(6.4)	0.8
Mycoplasma pneumoniae	1 (0.1)	1(0.1)	1.0				9 (0.2)	1(0.1)	2.0				2 (0.2)	0(0)	/			
Neisseria meningitides				3 (0.4)	4(0.2)	2.0				4 (0.1)	4(0.2)	0.5				1 (0.1)	2(0.6)	0.2
Parainfluenza virus type 1	2 (0.2)	7(0.4)	0.5				23 (0.6)	7(0.4)	1.5				3 (0.3)	4(1.0)	0.3			
Parainfluenza virus type 2	0	7(0.4)	/				7 (0.2)	7(0.4)	0.5				2 (0.2)	2(0.5)	0.4			
Parainfluenza virus type 3	8 (0.8)	16(0.6)	1.3				51 (1.3)	16(0.6)	2.2				14 (1.2)	6(1.5)	0.8			
Pseudomonas aeruginosa				6 (0.8)	7(0.4)	2.0				13 (0.4)	7(0.4)	1.0				2 (0.2)	3(0.9)	0.2
Respiratory syncytial virus	62 (6.4)	25(1.3)	4.9				331 (8.3)	25(1.3)	6.4				43 (3.7)	3(0.7)	5.3			
Rhinovirus / Enterovirus	223(22.8)	644(34.0)	0.7	24 (3.2)	49(2.9)	1.1	1185(29.8)	644(34.0)	0.9	105 (3.2)	49(2.9)	1.1	419(36.1)	190(46.1)	0.8	12 (1.4)	6(1.7)	0.8
Rubella	5 (0.5)	5(0.3)	1.7				15 (0.4)	5(0.3)	1.3				5 (0.4)	1(0.2)	2.0			
Salmonella sp.				14 (1.9)	30(1.8)	1.1				60 (1.8)	30(1.8)	1.0				21 (2.5)	8(2.3)	1.1
Staphyloccocus aureus				8 (1.1)	15(0.9)	1.2				21 (0.7)	15(0.9)	0.8				6 (0.7)	10(2.9)	0.2
Streptococcus pneumoniae	286(29.3)	628(33.2)	0.9	16 (2.1)	20(1.2)	1.75	1436(36.2)	628(33.2)	1.1	44 (1.4)	20(1.2)	1.2	593 (51.1)	248(60.2)	0.8	9 (1.1)	10(2.9)	0.4
Ureaplasma sp.	152(15.6)	118(6.2)	2.5	3 (0.4)	6(0.4)	1.0	397(10.0)	118(6.2)	1.6	14 (0.4)	6(0.4)	1.0	117 (10.1)	42(10.2)	1.0	2 (0.2)	2(0.6)	0.3

[1]NP/OP=Nasopharyngeal/oropharyngeal

[2]Critically Ill: NP/OP TAC: Number of cases tested for Cytomegalovirus N = 929; Number of controls tested for Cytomegalovirus N = 1800; Blood TAC: Number of cases tested for *Neisseria meningitidis* N = 744

[3]Clinical severe infection: NP/OP TAC: Number of cases tested for Cytomegalovirus N = 3580; Number of controls tested for Cytomegalovirus N = 1800; Number of cases tested for *Neisseria meningitidis* N = 3238

[4]Sylhet Fast Breathing Only: NP/OP TAC: Number of cases tested for Cytomegalovirus N = 1013; Number of controls tested for Cytomegalovirus N = 357; Number of cases tested for *Neisseria meningitidis* N = 844

Supplemental material 2. Characteristics of pathogens isolated from blood culture among infants with possible serious infection, by critically ill and clinical severe infection status, Aetiology of Newborn Infections in South Asia (ANISA) Study, 2011-14

**Pathogen**	**Critically Ill Infant pSBI episodes**	**Clinical Severe Infection Infant pSBI episodes**
Subtotal gram positive		
Enterococcus faecium	1	
Group A *Streptococcus*		11
Group B *Streptococcus*	2	4
Staphylococcus aureus	4	8
Streptococcus oralis		
Streptococcus pneumoniae	2	3
Subtotal gram negative		
Acinetobacter	2	1
Burkholderia cepacia	1	1
Citrobacter koseri		1
Edwardsiella tarda	1	
Enterobacter sakazakii	2	1
Escherichia coli	7	13
Klebsiella pneumoniae	10	6
Morganella morganii		1
Neisseria meningitidis	1	4
Plesiomonas shigelloides		1
Proteus mirabilis		1
Pseudomonas aeruginosa	1	
Pseudomonas pseudomallei	2	1
Salmonella enterica		2
Serratia marcescens	2	1
**Total**	**38**	**60**

Supplemental material 3. Characteristics of pathogens isolated from blood culture among infants with possible serious infection in Sylhet, by age of onset and fast breathing only status, Aetiology of Newborn Infections in South Asia (ANISA) Study, 2011-14

		**Early Onset**		**Late Onset**	
**Pathogen**	**Total Number**	**Sylhet Clinical Severe (n=523)**	**Sylhet Fast breather (n=473)**	**Sylhet Clinical Severe (n=702)**	**Sylhet Fast Breather (n=529)**
Subtotal gram positive					
Enterococcus faecium					
Group A *Streptococcus*	7	1	3	3	
Group B *Streptococcus*	5	5			
Staphylococcus aureus	1			1	
Streptococcus oralis	1	1			
Streptococcus pneumoniae	4	2		2	
Subtotal gram negative					
Acinetobacter					
Burkholderia cepacia					
Citrobacter koseri					
Edwardsiella tarda	1			1	
Enterobacter sakazakii	1				1
Escherichia coli	5	2	1	2	
Klebsiella pneumoniae	1	1			
Morganella morganii	1			1	
Neisseria meningitidis	4			4	
Proteus mirabilis					
Pseudomonas pseudomallei	1	1			
Salmonella enterica					
Serratia marcescens					
**Total**	**32**	**13**	**4**	**14**	**1**
